# Supplementary material for: Quantifying the impact of homophily and influencer networks on song popularity prediction
Source: Sci Rep. 2024 Apr 18;14:8929. doi: 10.1038/s41598-024-58969-w (PMC11026404; doi:10.1038/s41598-024-58969-w)
Supplement: Supplementary file 1 — Supplementary Information. [file 41598_2024_58969_MOESM1_ESM.pdf]

Supplementary information

A. SI 1: Influencers and followers

In Fig. S1, we report some heterogeneity of users in their influencing behavior. While some users tend to discover new songs early and (directly or indirectly) promote them in their friendship network –acting as “influencers”– others tend to follow these users and adopt their discoveries. We find that the higher the influence score of early song listeners, the quicker we can expect information about that song to spread in the friendship network. In the main text, we show how this information can improve machine learning models that predict the popularity of songs.

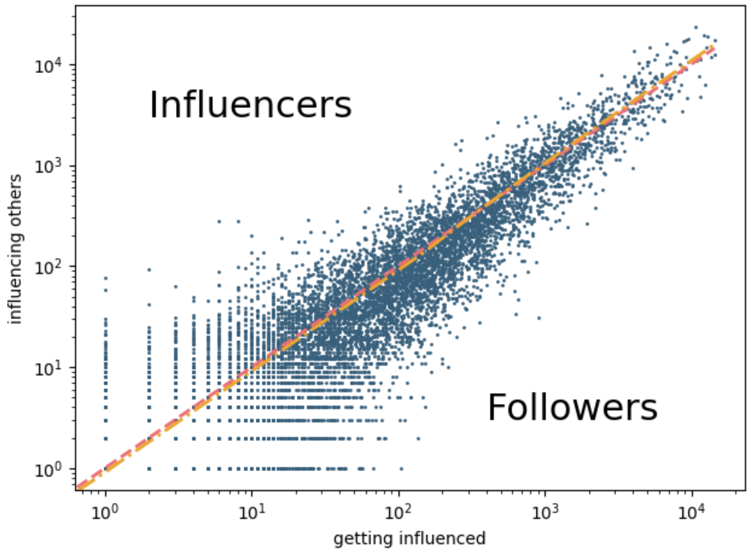

Figure S1. Influencing others vs getting influenced by others. Each dot represents a user. The number of times a user was influenced into listening to a song is marked on the x-axis. The number of times a user influenced another user is marked on the y-axis. The red dashed line marks x=y. The yellow dashed and dotted line marks a power-law fit with $\alpha = 1.009$. Users far above the red line tend to act as "influencers", while users far below tend to act as "followers".

B. SI 2: Homophily vs influence

In figure S2 we show a scatterplot of how the cosine similarity of users relates to their influence score.

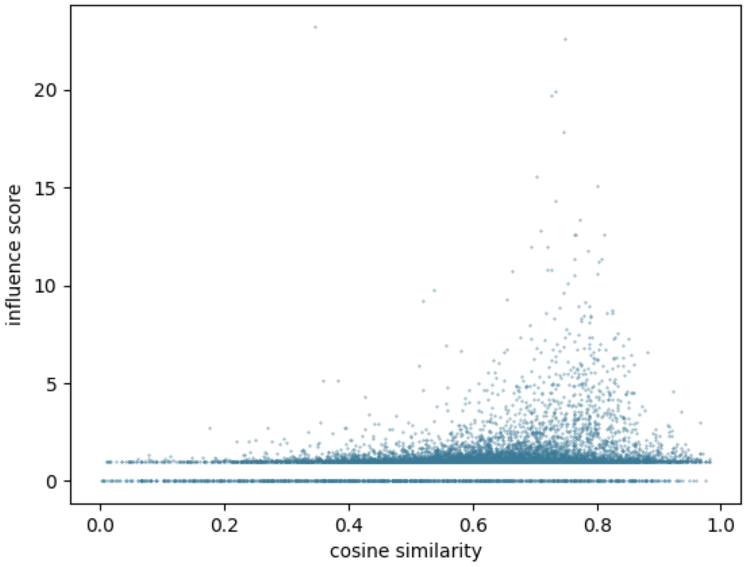

Figure S2. User influence score vs average cosine similarity between a user and all of their friends. Each dot represents a user. The apparent gap between zero and one is due to to normalization of the influence score.

C. SI 3: Classification vs regression

In our study aimed at determining the most effective approach for predicting song popularity, whether through regression or classification techniques, we implemented comparative analyses using both methodologies. Given the computational demands of processing the entire dataset, we opted to conduct our experiments on a randomly selected subset approximately one-third the size of the original dataset. This approach enabled us to maintain the integrity of our analysis while ensuring feasibility.

For the regression analysis, we constructed an ensemble model incorporating various algorithms and their respective hyperparameters, as follows: Support Vector Regression (SVR)[9] with a regularization parameter (C) set to 1; Random Forest Regression[4] with 200 estimators, a minimum sample split of 4 and no maximum depth; Ada Boost Regression[1] configured with 100 estimators and a learning rate of 0.01; Gradient Boosting Regression[2] utilizing 100 estimators, a learning rate of 0.1, and a maximum depth of 3; Histogram Gradient Boosting Regression[3] with a learning rate of 0.1 and maximum iterations set to 100; K-Neighbors Regression[7] with 7 neighbors; and Multi-Layer Perceptron (MLP) Regression[8], which was tuned with an alpha value of 0.01 and hidden layer sizes of (50, 50). All models were sourced from the scikit-learn library. These optimal hyperparameters for our models were identified by employing a grid search strategy (GridSearchCV)[6] coupled with 5-fold cross-validation, ensuring a robust tuning process. The chosen models were then integrated into a voting ensemble using the scikit-learn's VotingRegressor[5], promoting a collective decision-making process for the regression predictions. In our analysis, we adjusted the definition of a "hit" song to correspond with the scaled-down dataset, setting the threshold at a proportionate level to the original 1000-listenings benchmark which equates to 300 listenings.

Both the regression and classification ensembles were subjected to 20 training and evaluation cycles.

The mean absolute error (MAE), root mean squared error (RMSE), median absolute error (MEDAE), and coefficient of determination (r2) values of the regression were found to be 81.0, 148.2, 67.4, and 0.30, respectively. This indicates that the model successfully identifies certain patterns in predicting hit songs, yet it also implies that there are noteworthy factors influencing song popularity that have not been incorporated into the model. The fact that the Median Absolute Error (MEDAE) is significantly smaller than the Root Mean Squared Error (RMSE) or Mean Absolute Error (MAE), suggests that the distribution of errors in the predictions is skewed towards lower values, which is likely caused by the highly skewed distribution of song popularity itself. Figure S3 shows a scatter plot of the predicted values vs the data.

When statistically comparing the performance metrics, the means of accuracy, precision and recall deviated by 0.1%, 1%, and 2% respectively. However, independent samples t-tests, yielded p-values consistently above the 0.05 threshold, indicating no statistically significant differences between classification and regression results.

These findings suggest a parity in the viability of regression and classification approaches for predicting song popularity. The choice between these methods should, therefore, be guided by the specific requirements of the task at hand, including considerations related to data characteristics, optimization of hyperparameters, selection of loss functions, and the implementation

of feature engineering strategies.

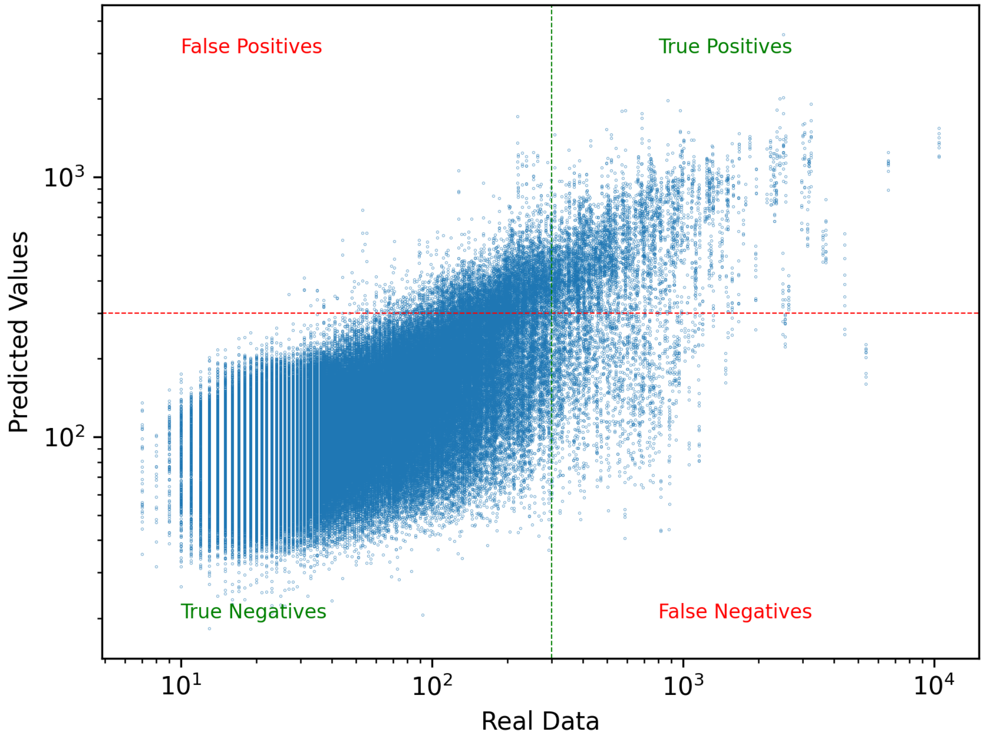

Figure S3. Regression results for predicting hit songs. The vertical green line denotes the hit song threshold in the dataset, distinguishing true hits (to the right) from average songs (to the left). The horizontal red line represents the prediction threshold for hit songs, indicating all predicted hits lie above it, while predicted average songs fall below.

References

1. scikit-learn developers. sklearn.ensemble.adaboostregressor. `https://scikit-learn.org/stable/modules/generated/sklearn.ensemble.AdaBoostRegressor.html`, 2024. Accessed: 2024-03-06.
2. scikit-learn developers. sklearn.ensemble.gradientboostingregressor. `https://scikit-learn.org/stable/modules/generated/sklearn.ensemble.GradientBoostingRegressor.html`, 2024. Accessed: 2024-03-06.
3. scikit-learn developers. sklearn.ensemble.histgradientboostingregressor. `https://scikit-learn.org/stable/modules/generated/sklearn.ensemble.HistGradientBoostingRegressor.html`, 2024. Accessed: 2024-03-06.
4. scikit-learn developers. sklearn.ensemble.randomforestregressor. `https://scikit-learn.org/stable/modules/generated/sklearn.ensemble.RandomForestRegressor.html`, 2024. Accessed: 2024-03-06.
5. scikit-learn developers. sklearn.ensemble.votingregressor. `https://scikit-learn.org/stable/modules/generated/sklearn.ensemble.VotingRegressor.html`, 2024. Accessed: 2024-03-06.
6. scikit-learn developers. sklearn.model_selection.gridsearchcv. `https://scikit-learn.org/stable/modules/generated/sklearn.model_selection.GridSearchCV.html`, 2024. Accessed: 2024-03-06.
7. scikit-learn developers. sklearn.neighbors.kneighborsregressor. `https://scikit-learn.org/stable/modules/generated/sklearn.neighbors.KNeighborsRegressor.html`, 2024. Accessed: 2024-03-06.

8. scikit-learn developers. sklearn.neural_network.mlpregressor. https://scikit-learn.org/stable/modules/generated/sklearn.neural_network.MLPRegressor.html, 2024. Accessed: 2024-03-06.

9. scikit-learn developers. sklearn.svm.svr. https://scikit-learn.org/stable/modules/generated/sklearn.svm.SVR.html, 2024. Accessed: 2024-03-06.
